# Protein Diet Restriction Slows Chronic Kidney Disease Progression in Non-Diabetic and in Type 1 Diabetic Patients, but Not in Type 2 Diabetic Patients: A Meta-Analysis of Randomized Controlled Trials Using Glomerular Filtration Rate as a Surrogate

**DOI:** 10.1371/journal.pone.0145505

**Published:** 2015-12-28

**Authors:** Mahesh Shumsher Rughooputh, Rui Zeng, Ying Yao

**Affiliations:** 1 Department of Nephrology, Tongji Hospital, Tongji Medical College, Huazhong University of Science and Technology, Wuhan, China; 2 Department of Clinical Nutrition, Tongji Hospital, Tongji Medical College, Huazhong University of Science and Technology, Wuhan, China; Emory University, UNITED STATES

## Abstract

**Background/ Objective:**

Studies, including various meta-analyses, on the effect of Protein Diet Restriction on Glomerular Filtration Rate (GFR) in Chronic Kidney Disease (CKD) have reported conflicting results. In this paper, we have provided an update on the evidence available on this topic. We have investigated the reasons why the effect has been inconsistent across studies. We have also compared the effect on GFR in various subgroups including type 1 diabetics, type 2 diabetics and non-diabetics.

**Method:**

We searched for Randomized Controlled Trials on this intervention from MEDLINE, EMBASE, and other information sources. The PRISMA guidelines, as well as recommended meta-analysis practices were followed in the selection process, analysis and reporting of our findings. The effect estimate used was the change in mean GFR. Heterogeneity across the considered studies was explored using both subgroup analyses and meta-regression. Quality assessment was done using the Cochrane risk of bias tool and sensitivity analyses.

**Results:**

15 randomized controlled trials, including 1965 subjects, were analyzed. The pooled effect size, as assessed using random-effects model, for all the 15 studies was -0.95 ml/min/1.73m^2^/year (95% CI: -1.79, -0.11), with a significant p value of 0.03. The combined effect estimate for the non-diabetic and type 1 diabetic studies was -1.50 ml/min/1.73m^2^/year (95% CI: -2.73, -0.26) with p value of 0.02. The effect estimate for the type 2 diabetic group was -0.17 ml/min/1.73m^2^/year (95% CI: -1.88, 1.55) with p value of 0.85. There was significant heterogeneity across the included studies (I^2^ = 74%, p value for Q < 0.0001), explained by major variations in the percentage of type 2 diabetic subjects, the number of subjects and overall compliance level to diet prescribed.

**Conclusion:**

Our findings suggest that protein diet restriction slows chronic renal disease progression in non-diabetic and in type 1 diabetic patients, but not in type 2 diabetic patients.

## Introduction

The effect of protein diet restriction on kidney disease progression in patients with chronic kidney disease (CKD) has long been a topic of controversy [1]. Clinical trials including CKD subjects or subgroups of this population have shown varying results.

Despite using the same outcome measure to assess kidney disease progression, that is change in mean GFR; previous meta-analyses have been dissimilar in their findings. For instance, while the meta-analysis of Yu Pan et al. [2] reported no significance of protein diet restriction, that reported by Uru Nezu et al. [3] found a significant benefit. Both have discussed the long term effect of this intervention in the diabetic patients having CKD. However, both meta-analyses showed a non-significant effect of protein diet restriction in the type 2 diabetic group.

Another discrepant aspect of these analyses is the amount of heterogeneity. Heterogeneity represents how uniform the studies are in a pooled analysis. Guidelines on methodology [4] and evidence grading bodies [5] have continuously discussed the importance of quantitatively reporting the amount of heterogeneity in a meta-analysis. There are two currently accepted units to measure such variations, namely: the chi-square test for heterogeneity (Q) and the variability due to heterogeneity (I^2^). In the meta-analysis done by Uru Nezu et al [3], the amount of heterogeneity was significant.

There is a need to find out why the results were inconsistent across the studies.

In this paper, we therefore report an updated meta-analysis, specifically, on the effect of protein diet restriction on GFR in the CKD population. We have only reviewed Randomized Controlled Trials (RCTs) describing this intervention. We have compared the results of the type 1 diabetic, type 2 diabetic and non-diabetic groups to determine whether the etiology for CKD influenced the effect of protein diet restriction. We have also explored other possible causes of inconsistency in the results of individual studies.

## Materials & Methods

This meta-analysis was planned, conducted and reported conforming to the Preferred Reporting Items for Systematic Reviews and Meta-Analysis (PRISMA) statement [6]. The Cochrane handbook [4] was used as a methodological reference. The selection criteria and the methods of the analysis were specified in advance (see S2 File for the study protocol).

### Study selection: Inclusion criteria and search strategy

The inclusion criteria were as follows:

- Studies reported in English or available as English translated articles
- Randomized controlled trials
- Study duration of more than 12 months, which is the time stated to detect permanent changes in GFR [7]
- Studies reporting change in mean GFR or reporting baseline and final mean GFR
- Studies limited to restriction of protein intake without supplementation with Essential Amino Acids (EAA) or Keto-Amino Acids (KAA) so as to enable the assessment of the effect of dietary protein restriction alone rather than the combined effect of both dietary restriction and supplementation

Studies reporting an intervention period of less than one year in either arm of their design, those not quantifying GFR, and those whose participants included patients on dialysis were excluded. We have also excluded all cross-over trials in our analysis. In a cross-over trial, multiple interventions are consecutively performed on the same group of subjects. There is the risk that the first intervention influences the outcome of the second intervention. The effect of the second intervention is not reflected independently. This is termed as carryover effect [8].

RCTs were searched from the MEDLINE database, the EMBASE database and the Cochrane Central Register of Controlled Trials (CENTRAL) since their inception till 24^th^ September 2014. We also retrieved studies from clinicaltrials.gov, numerous journals on nutrition and renal medicine, guidelines on CKD, reviews and from the reference lists of the already published articles. Key terms “chronic renal disease” AND “glomerular filtration rate” AND “protein diet” AND “restriction” AND “randomized controlled trial” with limits “human” were used with variations. We used specific Medical Subject Headings (MeSH) in our construct. We have supplemented the search strategy and a flowchart for the selection process from the MEDLINE database performed on 24/09/2014 in S3 File.

All identified studies were read independently by the authors, and a final list of included studies was made after thorough discussion.

### Data Extraction and Bias assessment

The baseline characteristics of each study and its participants were extracted. These included the subjects’ baseline GFR or estimated glomerular filtration rate (eGFR) or creatinine clearance, baseline proteinuria, baseline albuminuria, mean age of the participants, their gender distribution, their average weight, the percentage of diabetic patients, duration of diabetes of the diabetic subjects, their type of diabetes and the mean arterial pressure. Data were also extracted on the various quantitative measures to assess protein intake and the type of analysis made, that is, whether the report was an intention-to-treat analysis or an available case analysis.

To assess the risk of bias in individual studies, the Cochrane Collaboration’s risk of bias tool was used [9]. This tool rates bias of an RCT in three categories (low, unclear, and high) on the following domains: Random Sequencing, Allocation Concealment, Blinding of Participants, Blinding of Personnel, Blinding of Outcome Assessment, Incomplete Outcome Data, Selective Reporting Bias, and Other risk. The other risk of bias domain was categorized into high and low risk of bias depending on whether or not the Modification of Diet in Renal Disease (MDRD) equation was used to calculate eGFR in a particular study as recent guidelines [10, 11] have questioned the sensitivity of this equation, which is affected by the age of the patient and stage of renal disease. The overall score given to a study is the worst score achieved in any of these domains. Each study was independently assessed by 2 authors. The worse score in each field was reported. Blinding of participants and personnel was not considered for overall scoring risk of bias. The intervention of restricting protein intake itself involves the cooperation of the patient, and such blinding prevents a proper two-way rapport between dietician and patient. However, we did consider Blinding of Outcome assessment in scoring the studies.

### Statistical Analysis

The primary outcome was the change in mean GFR. We have alternatively used change in mean eGFR or mean creatinine clearance to calculate the effect estimate for some of the studies. All studies reported the primary outcome on the same scale and the reported values were almost normally distributed. When not primarily reported, we used the Follmann technique [12] to impute the change in mean GFR, using the final and initial (baseline) values for mean GFR.

The assessment of protein intake was based on reported urinary indices. The actual protein intake was derived using the Maroni equation [13], which is based on Urinary Urea Nitrogen (UUN), assuming nitrogen balance is maintained. The percentage of protein over-intake for each arm of a study was calculated. The measure derived to assess compliance level in a study, the compliance ratio, was calculated as the ratio of the percentage of protein over-intake in the experimental arm to that of the control arm.

The effect estimates from the studies were pooled together and analyzed using random-effects model, since we expected much heterogeneity. We quantified heterogeneity using the two currently accepted measures, namely: the chi-square test for heterogeneity (Q) and the variability due to heterogeneity (I^2^).

The risk of bias across studies was assessed visually using a funnel plot. We also conducted Egger Regression test [14] and Begg and Mazumdar Rank Correlation test [15] as formal statistical tests for publication bias.

Sensitivity analyses were performed on the type of data analysis (intention-to-treat analysis or available case analysis), on the studies that used the MDRD equation to calculate eGFR and on the studies that required imputation for the effect size calculations. These criteria were all defined before analysis.

Heterogeneity was explored using two methods, namely subgroup analysis and meta-regression. In subgroup analysis, we categorize the pooled studies into two or more subgroups, based on a predefined characteristic, with the aim of reducing heterogeneity. For subgroup analysis based on etiology for CKD, we grouped all studies done exclusively on type 2 diabetic patients together.

Meta-regression, on the other hand, is an established statistical tool used to assess the relationship between explanatory variables (potential effect modifiers) and an outcome variable (change in mean GFR). The explanatory variables are characteristics of study that may affect the effect estimate [4].

As predefined in the study protocol, we identified age, duration of study, percentage of type 2 diabetic subjects, compliance derived from actual protein intake, mean arterial pressure, baseline GFR, baseline proteinuria, and mean duration of diabetes as potential effect modifiers since these have all been confirmed as factors affecting GFR [16]. All potential effect modifiers were continuous outcomes. A random-effects meta-regression model was used to examine the contribution of these to heterogeneity and adjust for the effect estimate. The Z-test statistics was used to test for statistical significance of each potential effect modifier. The proportion of variance explained (R^2^) was used to quantify the amount of heterogeneity accounted for by each. It was calculated as the ratio of variance explained to the total amount of variance. A model with combination of these potential effect modifiers was then derived. The aim of creating a model was to lower heterogeneity; that is, to decrease the variability due to heterogeneity (I^2^) to the minimum possible value and to bring the p value for Q as close as possible to 1. The principle is that, in an ideal situation, heterogeneity is completely absent (I^2^ is 0 and p value for Q is 1) [17]. The assessment of the model fit (normality of residuals) was done using the normal probability plot. This plot is a graphical method for comparing two probability distributions by plotting their corresponding quantiles against each other [18].

The p value for significant heterogeneity was taken as less than 0.01. For all other comparisons, statistically significant differences were those with p-values of at most 0.05. All values were listed as mean (standard deviation) or estimate (lower limit of 95% confidence interval, upper limit of 95% confidence interval) unless specified.

We used R version 3.1.2 (2014-10-31) software [19] with the ‘metafor’ package version 1.9–5 [20], which has been validated for such analysis [21], to perform our statistical analysis.

## Results

### Description of included studies

**Fig 1:**
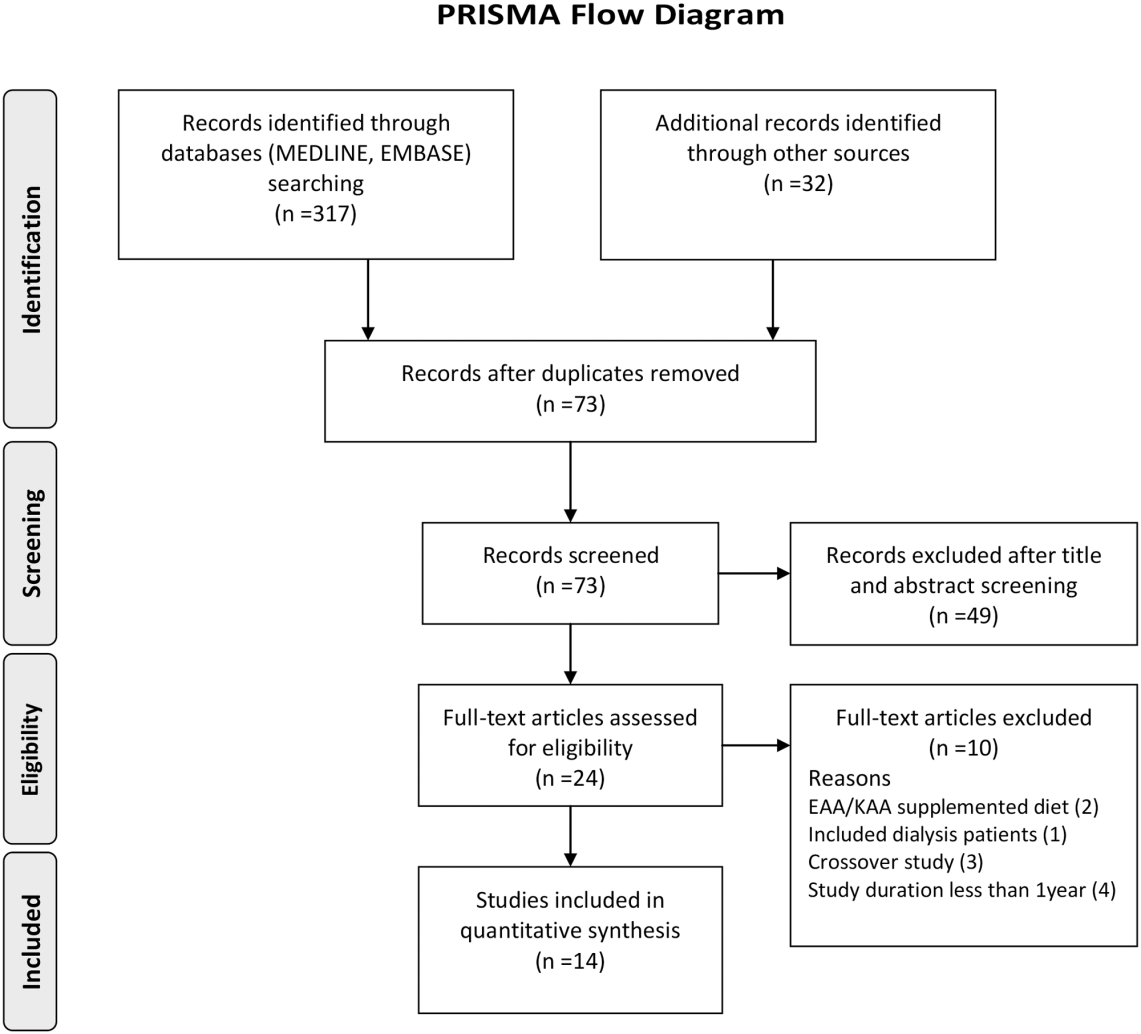
The PRISMA flow diagram for the study selection process. From: Moher D, Liberati A, Tetzlaff J, Altman DG, The PRISMA Group (2009). Preferred Reporting Items for Systematic Reviews and Meta-Analyses: The PRISMA Statement. PLoS Med 6(6): e1000097. doi:10.1371/journal.pmed1000097.

Twenty-four full text articles were assessed for eligibility. We excluded ten, namely three cross-over trials, two studies having protein restriction with amino acids supplementation as intervention, four articles having study duration of less than one year, and one report including patients on dialysis. We, thus, identified fourteen articles that met our selection criteria: fifteen studies taking Meloni et al. paper [22] as two separate studies, the trial on the diabetic subjects and that on the non-diabetic subjects. The PRISMA flow diagram illustrates the selection process throughout (Fig 1).

The characteristics of included studies are described in Table 1. Ten studies performed an Intention-to-Treat (ITT) analysis, three reported available cases, and two did not state the type of analysis. Of the 1965 subjects, 782 were diabetic and 1183 were non-diabetic. Six studies reported an average proteinuria of over 1g/24h; 7 stated the baseline albuminuria; and two did not quantify either. The population comprised mainly of hypertensive patients and most of the subjects were on Angiotensin Converting Enzyme (ACE) Inhibitors. Only four trials avoided use of ACE inhibitors [20, 21, 24, 30]. Two trials included exclusively non-diabetic subjects while three included mostly non-diabetic. Four studies reported exclusively on type 2 diabetics, four exclusively on type 1 diabetics, and two combined results from both the etiologies. The percentage of diabetic patients and the type of diabetes were directly stated in most studies. In those where these were absent, percentage of diabetes was calculated from the number of diabetic subjects in the study. Three studies did not report baseline mean for weight. Only one study performed exclusively on type 2 diabetic subjects failed to mention the average duration of diabetes. All the studies reported group data; no individual data was used to calculate any of the baseline characteristics. For each study, the mean values reported for the entire study group were extracted. The baseline characteristics were well balanced in either arms of each RCT included.

**Table 1:** Characteristics of the included studies ^a^.

Index	Author [ref]	PY	Study design	n	Baseline mean GFR (ml/min/1.73m^2^)	Baseline Proteinuria or Albuminuria^#^ (mg/24h)	Duration of study (months)	Mean Age (years)	Male (%)	Weight (kg)	DM (%)	Type of DM	Duration of DM (years)	MAP (mm Hg)
1	B.U.Ihle et al.[23]	1989		72	14		18	37	66	68	0			103
2	B.H.Brouhard et al.[24]	1990		15	81	481^#^	12	33	60		100	1	19	99
3	K.Zeller et al. [25]	1991	ACA	35	47	3625	35	34	60		100	1	22	104
4	P.S.Williams et al. [26]	1991	ACA	95	26	3223	19	45	66	70	13	mixed		110
5	R.P.F.Dullaart et al. ^b^[27]	1993	ITT	30	126	48^#^	24	41	90	76	100	1	21	94
6	S.Klahr et al. Study A^c^[28]	1994	ITT	585	39		26	52	60		3	2		98
7	H.P.Hansen et al. [29]	2002	ITT	82	68	705^#^	48	41	65	70	100	1	28	100
8	L.T.J.Pijls et al. [30]	2002	ITT	131	64	21^#^	28	58	58	81	100	2	7	99
9	C.Meloni et al. DM ^d^[22]	2004	ITT	80	45	2500	12	55	48	65	100	mixed	24	104
10	C.Meloni et al. NDM ^d^[22]	2004	ITT	89	47	1999	12	62	51	66	0			104
11	B.Dussol et al. [31]	2005	ITT	63	86	373^#^	24	58	83	79	100	mixed	18	99
12	D.Koya et al. [32]	2009	ITT	112	62	1150	60	57	58	63	100	2		97
13	B.Cianciaruso et al. [33]	2009	ITT	423	16	1670	32	61	57	73	12	2		97
14	N.R.Larsen et al. [34]	2011	ITT	108	71	41^#^	12	59	49	95	100	2	9	98
15	D.R.Jesudason et al. [35]	2013	ACA	45	94	89^#^	12	61	78	106	100	2	10	

The table summaries all the characteristics extracted for each study. The mean value for each baseline characteristic is reported. No individual data was used for any calculation. All studies reported grouped data.

^a^PY, Year of publication; n, number of subjects in study; GFR, Glomerular Filtration Rate; MAP, Mean Arterial Pressure; ITT, Intention-to-treat analysis; ACA, Available case analysis; All values rounded to nearest whole number.

^#^denotes Baseline Albuminuria, in mg/24h, instead of Baseline Proteinuria.

^b^R.P.F.Dullaart et al. [27] study group was included in this review because the number of subjects falling in the hyper filtrating group in each arm of the study were the same.

^c^For the Modification of Diet in Renal Disease (MDRD) study, only study A was included in this review since study B involved the supplementation of diet.

^d^For C. Meloni et al. study, the two subgroups, namely the diabetic group (DM) and the non-diabetic group (NDM) were analyzed separately.

The lower protein diet prescribed in the experimental arm of most of the studies was an iso-caloric protein restricted diet, favoring intake of high biological value protein. The others reported a non-significant alteration in calorie intake. The increase of unsaturated fat and dietary fiber compensated for the lowering of protein in the diet. Only one study reported calcium supplementation. In general, the control diet was unrestricted, whereby the subjects were allowed to continue their usual dietary practices. Many of the diabetic subjects received counseling as per the recommended guidelines for diabetes. Details on the protein regimes are shown in Table 2.

**Table 2:** Dietary protein intervention in both arms for each study and the assessment of compliance for each study ^a^.

Index	Author	Planned Intake (g/kg/24h)		Actual Intake based on urinary indices ^b^ (g/kg/24h)		Difference between the two groups of the study ^c^	Percentage protein over-intake (%)^d^		Compliance ^e^
		Exp	Control	Exp	Control		Exp	Control	
1	B.U.Ihle et al. [23]	0.4	0.8	0.65	0.80	S	62.6	7.0	8.9
2	B.H.Brouhard et al. [24]	0.6	1.0	0.71	1.07	S	18.3	7.0	2.6
3	K.Zeller et al. [25]	0.6	1.0	0.72	1.08	S	20.0	8.0	2.5
4	P.S.Williams et al. [26]	0.6	0.8	0.72	0.89	S	20.0	11.3	1.8
5	R.P.F.Dullaart et al. [27]	0.6	1.0	0.79	1.09	S	31.7	9.0	3.5
6	S.Klahr et al. Study A ^f^ [28]	0.6	1.3	0.70	1.13	S	20.7	-13.1	-1.6
7	H.P.Hansen et al. [29]	0.6	1.0	0.89	1.02	S	48.3	2.0	24.2
8	L.T.J.Pijls et al. [30]	0.8	1.0	1.10	1.14	NS	37.5	14.0	2.7
9	C.Meloni et al. DM [22]	0.8	1.2	0.86	1.24	S	7.5	3.3	2.3
10	C.Meloni et al. NDM [22]	0.6	1.2	0.67	1.54	S	11.7	28.3	0.4
11	B.Dussol et al. [31]	0.8	1.2	1.10	1.03	NS	37.5	-14.2	-2.6
12	D.Koya et al. [32]	0.8	1.2	1.00	1.00	NS	25.0	-16.7	-1.5
13	B.Cianciaruso et al. [33]	0.6	0.8	0.73	0.90	S	32.7	12.5	2.6
14	N.R.Larsen et al. [34]	15.0^g^	30.0 ^g^	18.90 ^g^	26.50 ^g^	S	26.0	-11.7	-2.2
15	D.R.Jesudason et al. [35]	55–70 ^h^	90–120 ^h^	0.93	1.02	S	55.1	4.8	11.5

^a^ Exp, experimental group of the study; Control, control group of the study; S, significant; NS, not significant.

^b^ Actual protein intake was derived using Maroni Equation based on Urinary Urea Nitrogen (UUN) assuming Nitrogen balance. The formula used was protein intake, in grams, is equal to 6.25 (UUN, in grams, + 0.031 body weight, in kilograms).

^c^ Statistical significance for the difference in actual protein intake between groups, using urinary indices, reported.

^d^ Percentage of protein over-intake calculated as: [(actual protein intake–planned protein intake)/planned protein intake]*100

^e^ Compliance ratio is calculated as the ratio of: percentage of over-intake in the experimental arm / percentage of over-intake in the control arm.

^f^ Actual protein intake extracted from another article [36] of the same team on the same study.

^g^ In N.R.Larsen et al. [34] study, values expressed as percentage of total energy in diet. These values were used to calculate percentage of protein over intake and then the compliance ratio since actual protein intake was not reported.

^h^ In D.R.Jesudason et al. [35] study, the range of planned protein intake reported in g/24h. The average value used to calculate percentage over-intake and compliance.

Pooling the mean values for all studies, the achieved protein intake was 0.83 (0.15) g/kg/day in the experimental arm and 1.07 (0.17) g/kg/day in the control arm, based solely on urinary indices. The summary of all characteristics considered in our analyses is described in Table 3. The table reports median and interquartile range for skewed distributions while mean and standard deviations for normal distributions. As we can note, the distributions were skewed for the following parameters: number of subjects; baseline proteinuria; percentage of type 2 diabetic subjects, percentage of hypertensive patients, protein over-intake; and compliance ratio. However, there was no substantial skewness for the baseline GFR, study duration, weight, age, percentage of male, mean arterial pressure, and duration of diabetes.

**Table 3:** Summary of the characteristics of the population pooled from the included studies.

Characteristic	Unit	Mean OR (Median)	Standard Deviation OR (Inter-Quartile Range)
Number of subjects (n)		(131)	(152)
Baseline mean GFR	ml/min/1.73m^2^	62	35
Baseline proteinuria or albuminuria	mg/24hr	(1225)	(1217)
Duration of study	months	24	18
Age of subjects	years	55	18
Percentage of male subjects	%	60	8
Weight of subjects	kg	71	12
Percentage of Type 2 diabetic subjects in study	%	(40)	(45)
Average duration of diabetes	years	19	12
Percentage of hypertensive subjects	%	(80)	(29)
Average of Mean Arterial Pressure(MAP)	mm Hg	99	6
Planned protein intake in experimental group	g/kg/24hr	(0.6)	(0.2)
Planned protein intake in control group	g/kg/24hr	(1.0)	(0.2)
Percentage protein over-intake in the experimental group	%	(26.0)	(17.5)
Percentage protein over-intake in control group	%	(7.0)	(15.0)
Compliance ratio		(2.5)	(3.6)

The score for bias under the Random Sampling domain was low in all the studies. Five studies failed to state the method for Allocation Concealment. The score for Blinding of Outcome Assessment was unclear in one study. Only 2 included studies used the MDRD equation to calculate GFR. The overall score for the studies were as follows: 7 low risk, 5 unclear risk, and 2 high risk for bias. Hence, the overall risk of bias using the Cochrane risk of bias tool was low. The details of the bias assessment can be found in S4 File.

### Meta-analysis, Subgroup and Sensitivity Analyses

Two studies required imputation of effect size, calculated from reported final and baseline values. In all studies, mean GFR was reported in the appropriate unit (ml/min/1.73m2 BSA/year), and, as such, needed no conversion. For only one study, namely Williams P.S. et al [26], the change in mean GFR could not be derived. We used the reported change in mean creatinine clearance instead as its effect size.

**Fig 2:**
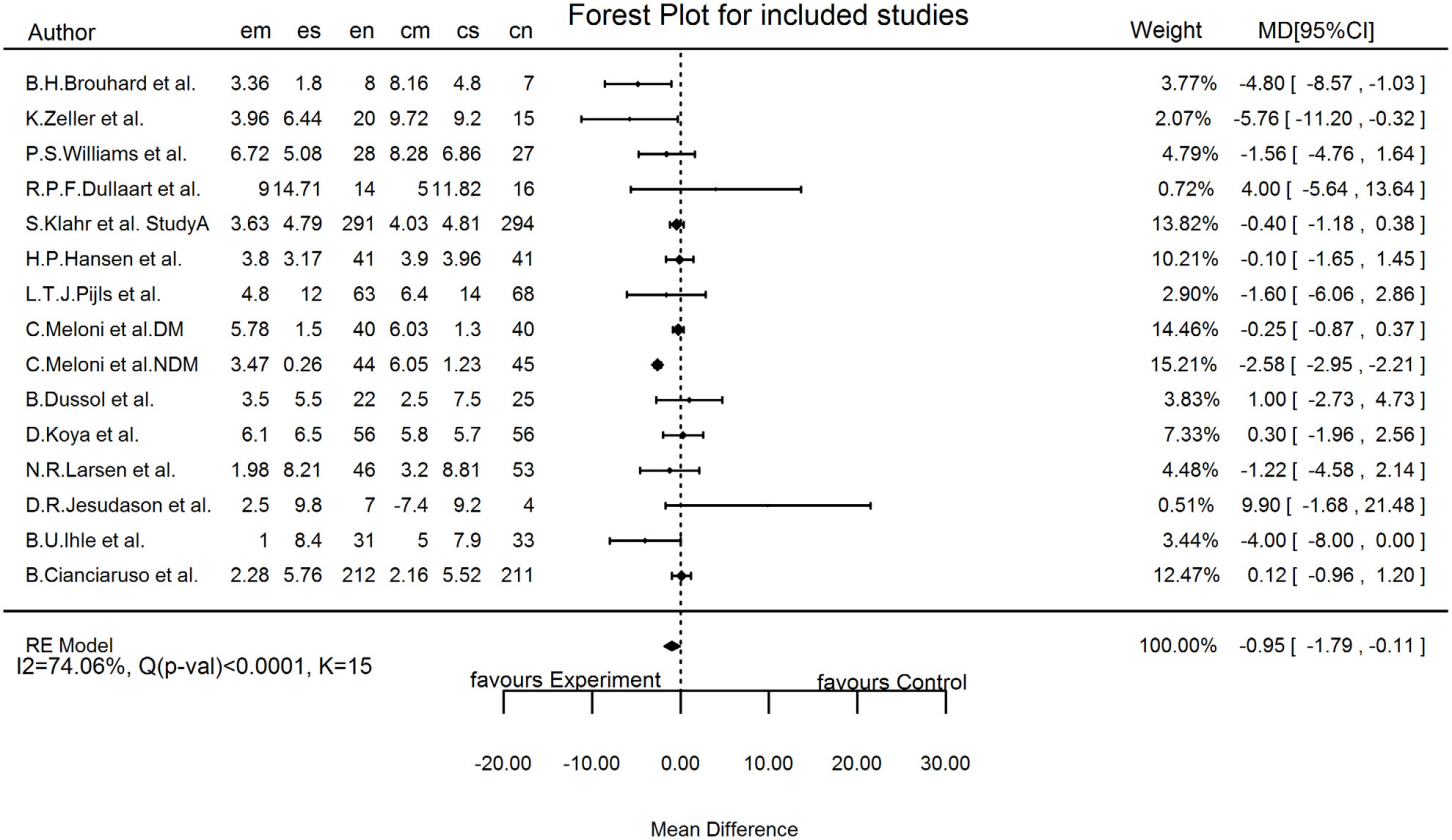
Forest Plot of all the included studies ^a^. The forest plot for all included studies pooled together using a random-effects model. ^a^ em, mean decline in experimental group; es, standard deviation in experimental group; en, number of subjects in experimental group; cm, mean decline in control group; cs, standard deviation in control group; cn, number of subjects in control group, MD, mean difference; I2, variability due to heterogeneity; Q, chi-square test; K, number of included studies.

The pooled effect size, as assessed using random-effects model, for all the 15 studies was -0.95 ml/min/1.73m^2^/year (-1.79, -0.11), with a significant p value of 0.03. There was significant heterogeneity, as assessed by both I^2^ (74.06%) and Q (p value < 0.0001). The forest plot (Fig 2) illustrates the pooled result and the relative contribution of each study in this pooled analysis.

Visually, the funnel plot was almost symmetrical, indicating minimal publication bias across the studies. It is supplemented in S1 Fig. The Egger Regression test (z = 0.09, p value = 0.93) and Begg and Mazumdar Rank Correlation test (Kendall’s tau = -0.28, p value = 0.17) were also not significant for bias.

Table 4 summarizes the subgroup and sensitivity analyses. Three studies could not achieve significant difference in protein intake between the experimental and control group. The combined effect size for these studies was also not significant. Subgrouping based on studies with type 2 diabetes as etiology of CKD homogeneously revealed a non-significant (p = 0.85) effect size of -0.17 ml/min/1.73m^2^/year (-1.88, 1.55); I^2^ = 0%; Q (p value) = 0.28. The effect estimate of the remaining studies; that is those including non-diabetics and type 1 diabetics, was significant (p value = 0.0175). The effect was -1.50 ml/min/1.73m^2^/year (-2.73, -0.26) with significant heterogeneity: I^2^ = 83%, Q (p value) < 0.0001. The results for the diabetic group of Meloni et al. [22] report was excluded in this analysis since it reported combined results for subjects of type 1 and type 2. We also sub-grouped the studies according to the baseline mean GFR into two groups, namely those more than or equal to 60 ml/min/1.73m^2^ BSA (stage G1 and G2) and those less than 60 ml/min/1.73m^2^ (stage G3 and 4). The former group showed no significant change in the effect estimate while the latter group had an effect estimate of -1.26 ml/min/1.73m^2^/year (-2.41, -0.11) with significant heterogeneity associated: I^2^ = 88%, Q (p value) <0.0001. Subgrouping according to mean age showed a significant effect size in the younger group. The six studies having a study duration of more than 2 years failed to reveal any significant result. Only five studies had more than 100 subjects and the effect size was not significant.

**Table 4:** Results of the subgroup and sensitivity analyses ^a^.

Parameter	Subgroup	K	Effect Estimate (ml/min/1.73m^2^/year)	Lower limit CI	Upper limit CI	Sig. estimate	I^2^ (%)	Q (p value)
Difference in Protein diet between group	Significant	12	-1.15	-2.11	-0.18	S	80	S
	Not significant	3	0.16	-1.62	1.93	NS	0	NS
Etiology	T2DM	4	-0.17	-1.88	1.55	NS	0	NS
	excl. T2DM	9	-1.50	-2.73	-0.26	S	83	S
Study duration (months)	>24	6	-0.25	-0.81	0.30	NS	0	NS
	≤24	9	-1.47	-2.79	-0.14	S	79	S
GFR	≥60	8	-0.35	-1.40	0.70	NS	0	NS
	<60	7	-1.26	-2.41	-0.11	S	88	S
Age (years)	>40	12	-0.59	-1.42	0.24	NS	75	S
	≤40	3	-4.69	-7.14	-2.24	S	0	NS
	>45	9	-0.61	-1.58	0.35	NS	81	S
	≤45	6	-2.28	-4.44	-0.12	S	51	NS
Number of subjects in study (n)	>100	5	-0.24	-0.83	0.35	NS	0	NS
	≤100	10	-1.45	-2.77	-0.12	S	82	S
Design of study	ITT	10	-0.59	-1.45	0.27	NS	79	S
	excl. ITT	5	-3.17	-5.21	-1.14	S	8	NS
sensitivity of MDRD	excl. MDRD use	13	-1.17	-2.08	-0.26	S	75	S
	MDRD use only	2	3.24	-5.69	12.18	NS	63	NS
Imputation for primary effect size	excl. imputations	13	-0.88	-1.72	-0.04	S	76	S
	Imputation only	2	-1.25	-8.68	6.19	NS	56	NS

^a^ K, number of studies included in analysis; CI, 95% confidence interval; Sig. estimate, significance of effect estimate; I^2^, variability due to heterogeneity; Q, chi-squared test for heterogeneity; S, significant; NS, not significant; T2DM, Type 2 Diabetes Mellitus; ITT, Intention-to-treat analysis.

Neither the exclusion of the 2 studies with imputation nor the exclusion of both studies which used the MDRD equation to calculate estimated GFR affected the significance of overall effect size. However, combined effect size for all the ITT results was not significant, with significant heterogeneity. The detailed result of analysis has been supplemented in S5 File.

### Meta-regression and validation of the model

Table 5 summarizes the results for the meta-regression analysis. We reported the intercept along with its confidence interval as well as the significance of the slope using Z-test statistics. We also included the proportion of variance explained (R^2^). We started by using one potential effect modifier at a time, as listed in the Material & Methods section. Though some of these corrected I^2^, none of these corrected for Q. Moreover, the p value for the effect of each was not significant. There was no significant contribution of baseline GFR or baseline proteinuria or Mean Arterial Pressure (MAP) as potential effect modifier in this analysis. We used combinations of the potential effect modifiers in increasing number to maximally correct for heterogeneity. The full stepwise analysis has been supplemented in S5 File.

**Table 5:** Summary of the results of the meta-regression analyses ^a^.

Grouping of studies	Potential effect modifier(s)	K	Intercept	lower CI	Upper CI	S est. ^b^	I^2^/ Q ^c^	R^2^	S mod ^b^
None	n, percentage of Type 2 DM, compliance	14	-3.10	-3.55	-2.64	S	NS	100	S
	n, percentage of Type 2 DM, compliance, age, study duration	14	-7.24	-12.07	-2.42	S	NS	100	S
Excl. T2 DM	n, study duration	9	-3.70	-4.35	-3.05	S	NS	100	S
NDG	study duration	5	-4.31	-5.09	-3.53	S	NS	100	S
T1 DM	age	4	-29.13	-48.21	-10.05	S	NS	100	S
T2 DM	compliance	4	0.12	-1.69	1.93	NS	NS	0	NS

^a^ K, number of studies included in analysis; CI, confidence interval; S est., significance of effect estimate; I^2^, variability due to heterogeneity; Q, chi- squared test for heterogeneity; R^2^, amount of heterogeneity accounted for; S mod, significance of moderator(s) in the model; T2 DM, Type 2 Diabetes Mellitus; NDG, non-diabetic group; T1 DM, Type 1 diabetes mellitus; S, significant; NS, not significant; n, number of subjects in the study.

^b^ The p value for significance of pooled effect estimate and potential effect modifier were taken as less than 0.05 (two tailed).

^c^ The p value for significant heterogeneity is less than 0.01.

Three potential effect modifiers explained most of the heterogeneity using the random-effects meta-regression model. These were the percentage of type 2 diabetics, compliance ratio, and number of subjects in each study. The proportion of variance explained (R^2^) was 100%. The best model further included study duration and mean age of subjects in the study. The potential effect modifier having the most influence in this model was the percentage of subjects having type 2 diabetes.

To assess the validity of the model, we pooled the non-diabetic population together. We observed that heterogeneity was substantially explained (R^2^ = 100%) by correcting for variations in study duration (one of the potential effect modifiers from the model). Similarly, we analyzed the diabetic group, subdividing it into those having type 1 and those having type 2. In the former group, the use of mean age (again, one of the potential effect modifiers from the model) corrected for heterogeneity (R^2^ = 100%). The effect size became significant. In the type 2 diabetic group, the result for effect size was already not significant without significant heterogeneity. Thus, any of the potential effect modifiers, including compliance ratio, only approached Q to 1.0 but none changed the result of effect size significantly. The normal probability plots for the random-effects model (no use of potential effect modifiers) and that of the random-effects meta-regression model (use of potential effect modifiers) are supplemented in S2 Fig.

## Discussion

Our results indicate that restriction of protein diet is beneficial in CKD subjects without diabetes or with diabetes type 1. However, this intervention does not delay decline in renal function in the type 2 diabetic group. Our results also show, with minimal heterogeneity, that protein diet restriction is not beneficial to patients with GFR more than 60 ml/min/1.73m^2^ BSA. The studies included in this meta-analysis had major variations in the percentage of type 2 diabetic subjects, the number of subjects and overall compliance level to diet prescribed. The 4 RCTs including exclusively type 2 diabetes subjects had a non-significant effect estimate with minimal heterogeneity. For the 4 reports on type 1 diabetes population, the effect estimate was significant with heterogeneity explained by age and for the 5 studies discussing mainly non-diabetic subjects, the effect estimate was significant, and heterogeneity explained by variations in study duration.

To our knowledge, 5 meta-analyses exist on the topic, namely Pan Y. et al. [2], Nezu U. et al. [3], Kasiske B.L. et al. [37], Robertson L. et al. [38] and Pedrini M.T. et al. [39]. Discrepancy was noted in their inclusion criteria. For instance: Kasiske B.L. et al. [37], Robertson L. et al. [38] and Pedrini M.T. et al. [39] included studies of different designs, namely RCTs and cross-over trials, in their analyses. As highlighted in our methods section, cross-over design suffers from the carry-over effect. This fact was illustrated by the meta-analysis of Kasiske B.L. [37], whereby it was found that the treatment effect for the included RCTs was less than that of the included non-randomized studies. Cross-over trials tend to overestimate the effect for such interventions. Another difference noted was that these analyses considered studies of less than one year which might not be sufficient to reveal permanent changes in GFR. These could explain their varying results though they have used change in GFR as effect measure. Interestingly, part of our results did coincide: Pedrini M.T. et al. [39] also reported a significant effect estimate for the type 1 diabetic nephropathy with no significant heterogeneity; Pan Y. et al. [2] and Nezu U. et al. [3] found a non-significant treatment effect for the type 2 diabetic subjects with CKD with significant heterogeneity in the latter analysis. Two RCTs, namely those conducted by G. D’Amico et al. [40] and Rosman J.B. [41], have also reported significant benefit of protein diet restriction in the non-diabetic group of CKD. However, due to missing data on the primary outcome, we could not include these in our analyses.

In a recent analysis involving type 2 diabetic subjects, Schwingshackl L. and Hoffmann G. [42] have also shown that protein diet restriction was not beneficial. On the contrary, they found that a higher protein diet significantly increased GFR and thus delayed kidney damage progression. Though our statistical methods were similar, their analysis combined studies on subjects without CKD and those in the early stages of the disease (GFR more than 60ml/min/1.73m^2^ BSA). Clinical trials of at least one week intervention period were considered, which might not be sufficient to show permanent changes in GFR. Their analysis involved both cross-over and randomized controlled trials. These differences might explain their bigger effect estimate in the opposite direction (in favor of a higher protein diet), a small significant p-value and bigger value for heterogeneity (I^2^) as compared to our results on the type 2 diabetic group with CKD.

Our study differed from the previous meta-analyses in the following: only RCTs were considered, only trials of more than one year were included, and new studies were included. We were able to find the true effect of protein restriction on GFR by using a justified inclusion criteria for study selection and limiting imputation to calculate effect size. We had a low risk of bias, for both across and in the studies included. Our sensitivity analyses showed change in neither significance nor direction of effect size. We used the two current methods to explore sources of heterogeneity, namely subgroup analysis and meta-regression. The results of these two analyses were similar. We made use of meta-regression techniques, which added robustness to the analysis of potential effect modifiers. The use of each potential effect modifier on a continuous scale avoided subjectivity in categorizing data.

The number of studies available in this analysis was the major limitation. The non-diabetic group of CKD patients includes a diversity of etiologies. A quantitative analysis of the latter was not possible. We would expect that some of its subgroups would benefit more from a restricted protein diet than others. Another limitation is that we have used changes in either mean GFR, or mean eGFR, or change in creatinine clearance interchangeably to calculate our effect size. Similarly, we have grouped both proteinuria and albuminuria as one for the analyses. Combining different methods for assessing a measure does add error in calculation of the effect size. We were not able to make a comparative analysis of each stage of kidney disease, using either baseline GFR or baseline albuminuria or baseline proteinuria. This was because the studies included combinations of different stages, without reporting the results in each stage or sub-stage individually. This prevented us from identifying at which stage of kidney disease the intervention of restricting protein intake has the most effect.

In this paper, we specifically explored the effect of protein diet restriction on change in GFR. Ideally, a complete balanced assessment for effect of protein diet restriction in CKD warrants consideration of other criteria such as adverse effects, cost and other renal parameters.

We not only restricted ourselves to the data analysis, but attempted to associate its significance to guide comprehension and further clinical work in the topic. We also illustrated that the number of subjects and duration of study are important design factors to be considered in planning such a clinical study. The calculation of the number-to-treat (NTT) is a crucial step in setting up of such an interventional trial. The duration of study, on the other hand, depends on the dynamics of the parameter being used to assess the effect.

It has been argued that change in GFR is a soft surrogate to renal disease progression [36]. It is less valid than other long term renal outcomes such as End-stage Renal Disease (ESRD) [43]. With sufficient time interval however, GFR reveals permanent changes to the kidney [44] rather than dynamic changes. The factors affecting GFR can be rightfully addressed by a planned RCT with balance in confounding factors between control and experimental groups. The importance of such planning in a design has already been detailed out by Consolidated Standards of Reporting Trials (CONSORT) group [45]. There is, however, a need to report results of a trial based on the causes for the CKD, rather than the combined result. This goes in line with the recommendation made by the recent Kidney Disease Improving Global Outcomes (KDIGO) guidelines [10] to classify CKD according to cause. This practice will allow the effect of an intervention to be directly compared. Our report illustrated that reporting of combined results may be a cause for inconsistency: the meta-regression analysis showed that the percentage of type 2 diabetes with CKD in each study affected the change in mean GFR.

In this analysis, we showed that age, as well as compliance, is important for selecting subjects who will benefit from a protein diet restriction in CKD. The impact of age in the type 1 diabetic group is clearly apparent. We can infer that the younger patients would benefit more from such intervention.

## Conclusion

Our comparative analysis shows that the restriction of protein in diet slows the chronic kidney disease progression in the non-diabetic group and the type 1 diabetic group of CKD significantly. In contrast, there is no benefit of restricting protein in diet in the type 2 diabetics with CKD.

The studies included differed in the number of subjects, percentage of type 2 diabetics, and level of compliance to the intervention. These factors explained the discrepancies in the individual results.

## Supporting Information

S1 File The PRISMA checklist. (PDF)

S2 File The protocol for the study. (PDF)

S3 File The search strategy and flow diagram for study selection from the MEDLINE database. (PDF)

S4 File The risk of bias assessment using the Cochrane Risk of Bias Tool. (PDF)

S5 File The spread-sheet of results. (XLSX)

S1 Fig Funnel Plot. The plot is almost symmetrical with 7 studies on one side and 8 on the other.
(TIF)

S2 Fig Probability plots for the random-effects model and random-effects meta-regression models. The left graph represents the probability plot for the random-effects model (no use of potential effect modifiers) and the right graph represents the random-effects meta-regression model (with use of the potential effect modifiers). The solid line represents the predicted effect for the normal distribution and the each dot represents a study. There is little organization in the distribution of dots in the random-effects model, with some close to the boundaries. In the plot for the random-effects meta-regression model with use of potential effect modifiers, it can be seen that the dots are more close to the predicted line. A normal distribution is achieved. The dots are arranged in a fairly straight line.
(TIF)
