# Supplementary material for: Protein Diet Restriction Slows Chronic Kidney Disease Progression in Non-Diabetic and in Type 1 Diabetic Patients, but Not in Type 2 Diabetic Patients: A Meta-Analysis of Randomized Controlled Trials Using Glomerular Filtration Rate as a Surrogate
Source: PLoS One. 2015 Dec 28;10(12):e0145505. doi: 10.1371/journal.pone.0145505 (PMC4692386; doi:10.1371/journal.pone.0145505)
Supplement: S2 File — (PDF) [file pone.0145505.s002.pdf]

Title

Protein Diet Restriction slows Chronic Kidney Disease Progression in non-diabetic and in type 1 diabetic patients, but not in type 2 diabetic patients: A meta-analysis of Randomised Controlled Trials using Change in Glomerular Filtration Rate as a surrogate

Date

August 2014

Team members

M.S.Rughooputh (first author & corresponding author)

Rui Zeng (corresponding author)

Ying Yao (corresponding author)

Abbreviations

CKD- Chronic Renal Disease

GFR- Glomerular Filtration Rate

eGFR- estimated Glomerular Filtration Rate

RCT- Randomised Controlled Trial

MeSH- Medical Subject Headings

MD- Mean Difference

SMD- Standardised Mean Difference

PRISMA- Preferred Reporting in Systematic Reviews and Meta-analysis

EAA- Essential Amino Acids

KAA- Keto Amino Acids

Background

- It is still debated whether or not we should implement protein restriction in diet in CKD to delay the progression of renal disease [1,2].
- The results of the various trials till date are not uniform in their explanation.
- The two most recent meta-analyses in the diabetic population have given opposing conclusions [3, 4].
- The others have also been non uniform in their conclusions, be it be in the non-diabetic group or the diabetic group.
- Amount of heterogeneity was different in all these analyses.
- GFR varies according to many factors [5].

Objectives

- Using change in mean GFR to get an updated answer
- Primary aim: Is Protein restriction beneficial in CKD?
- Secondary answers:
    - Is etiology a possible cause for inconsistency?
    - Is the result in type1 and type2 diabetic group of CKD the same?
    - In which subgroup is it beneficial?
    - Why have results been discrepant?
    - Find confounding factors having impact on results

Criteria of study

- Studies: RCT design, since randomization controlled trials tend to nullify the other factors affecting GFR
- Participants: CKD broad, since efficacy might be only in a particular group
- Intervention: All studies comparing a normal diet to one containing less protein
- Outcome measure: the change in GFR during the study period

Search strategy for identification of studies

- Aim at including the maximum number of RCTs.
- Search the main databases: MEDLINE and EMBASE
- Other search: Cochrane database (CENTRAL), clinicaltrials.gov, individual online journals, guidelines, reviews, email authors, explore conferences
- Fine search: references of articles
- Using MeSH to refine search

Method of review

- Use of Cochrane handbook[6] as primary guide to the analysis
- Limit imputation to increase quality
- Meta-analysis of data to detect heterogeneity
- Differential analysis based on etiology for CKD
- Use of potential effect modifiers– those that are factors affecting GFR (compliance, age, sex, BP, hypertension, diabetes, weight, smoking, and proteinuria) to explain heterogeneity.
- Use of subgroup and meta-regression [7] to correct for heterogeneity
- Subgroup analysis based on the results to validate findings

To ensure quality of analysis:

- Limit imputation
- Bias assessment using Cochrane risk of bias tool
    - Sensitivity analysis if significant discrepancy in bias
- Conform to PRISMA statement[8,9]
    - Organized way and recording of searches

Technical details

- Use of spread-sheet to collect data
- Analysis using R software

Inclusion criteria

- In English or translated fully
- RCTs
- Study duration of more than 1 year- to detect permanent changes to GFR [10]
- Quantify GFR decline- MD/ SMD depending on scale and most commonly reported method in the articles
- Quantify protein intake- use of objective method: example based on urinary indices- Maroni equation [11]

Exclusion criteria:

- Not true RCTs: imbalance in confounding factors
- Not quantifying the change in GFR decline
- Not stating the actual protein intake
- Not supplementing diet with EAA/KAA
- Not including patients undergoing dialysis

Proposed study Time frame

Duration (months)	Work to be done
1-2	Finalise protocol
1-2	Search for articles
2-3	Extract data
2-3	Analysis of data
1-2	Synthesis of report

Meetings: planned monthly to discuss issues and weigh progress

Funding

National Natural Science Foundation of China (81170686) and Research Project of Department of Health and Family Planning Commission, Hubei Province (WJ2015MB011)

References

1. Denis Fouque and William E. Mitch Low-protein diets in chronic kidney disease: are we finally reaching a consensus?Nephrol. Dial. Transplant. (2015) 30 (1): 6-8 first published online October 30, 2014doi:10.1093/ndt/gfu340
2. David W Johnson: Dietary protein restriction as a treatment for slowing chronic kidney disease progression: The case against (Review Article) Nephrology Volume 11, Issue 1, pages 58–62, February 2006 Article first published online: 1 MAR 2006 DOI: 10.1111/j.1440-1797.2006.00550.x
3. Nezu U, Kamiyama H, Kondo Y, Sakuma M, Morimoto T, Ueda S.: Effect of low-protein diet on kidney function in diabetic nephropathy: meta-analysis of randomised controlled trials. BMJ Open. 2013 May 28;3(5). pii: e002934. doi: 10.1136/bmjopen-2013-002934. PMID: 23793703
4. Pan Y, Guo LL, Jin HM.: Low-protein diet for diabetic nephropathy: a meta-analysis of randomized controlled trials. Am J Clin Nutr. 2008 Sep;88(3):660-6. PMID: 18779281Klahrfactors
5. Klahr S, Morrissey J.: Progression of chronic renal disease. Am J Kidney Dis. 2003 Mar; 41 (3 Suppl 1):S3-7.
6. Higgins JPT, Green S (editors). Cochrane Handbook for Systematic Reviews of Interventions Version 5.1.0 [updated March 2011]. The Cochrane Collaboration, 2011. Available at: www.cochrane-handbook.org.
7. Simon G. Thompson and Julian P.T. Higgins. How should meta-regression analyses be undertaken and interpreted? Statist.Med.2002; volume 21:pages 1559– 1573 [DOI:10.1002/sim.1187]

MRCBiostatisticsUnit;InstituteofPublicHealth;RobinsonWay;CambridgeCB22SR;U.K.

8. Moher D, Liberati A, Tetzlaff J, Altman DG, The PRISMA Group (2009). Preferred Reporting Items for Systematic Reviews and Meta-Analyses: The PRISMA Statement. PLoS Med 6(6): e1000097. doi:10.1371/journal.pmed1000097
9. Liberati A, Altman DG, Tetzlaff J, Mulrow C, Gøtzsche PC, et al. (2009) The PRISMA Statement for Reporting Systematic Reviews and Meta-Analyses of Studies That Evaluate Health Care Interventions: Explanation and Elaboration. PLoS Med 6(7): e1000100. doi:10.1371/journal.pmed.1000100
10. Gabriel Mircescu, Liliana Gârneaţă, Simona Hildegard Stancu, Cristina Căpuşă: Effects of a Supplemented Hypoproteic Diet in Chronic Kidney Disease. Journal of Renal Nutrition Volume 17, Issue 3, May 2007, Pages 179–188
11. Maroni BJ, Steinman TI, Mitch WE.: A method for estimating nitrogen intake of patients with chronic renal failure. Kidney Int. 1985 Jan;27(1):58-65. PMID: 3981873
