# Supplementary material for: Protein Diet Restriction Slows Chronic Kidney Disease Progression in Non-Diabetic and in Type 1 Diabetic Patients, but Not in Type 2 Diabetic Patients: A Meta-Analysis of Randomized Controlled Trials Using Glomerular Filtration Rate as a Surrogate
Source: PLoS One. 2015 Dec 28;10(12):e0145505. doi: 10.1371/journal.pone.0145505 (PMC4692386; doi:10.1371/journal.pone.0145505)

Flowchart for study selection from MEDLINE database

Date: 24/09/2014

Search performed:

("protein diet"[All Fields] OR "diet, protein"[All Fields] OR "intake, protein"[All Fields]) AND ("renal"[All Fields] OR "kidney function"[All Fields] OR "gfr"[All Fields] OR "glomerular filtration rate"[All Fields]) AND (Randomized Controlled Trial[ptyp] AND "humans"[MeSH Terms])

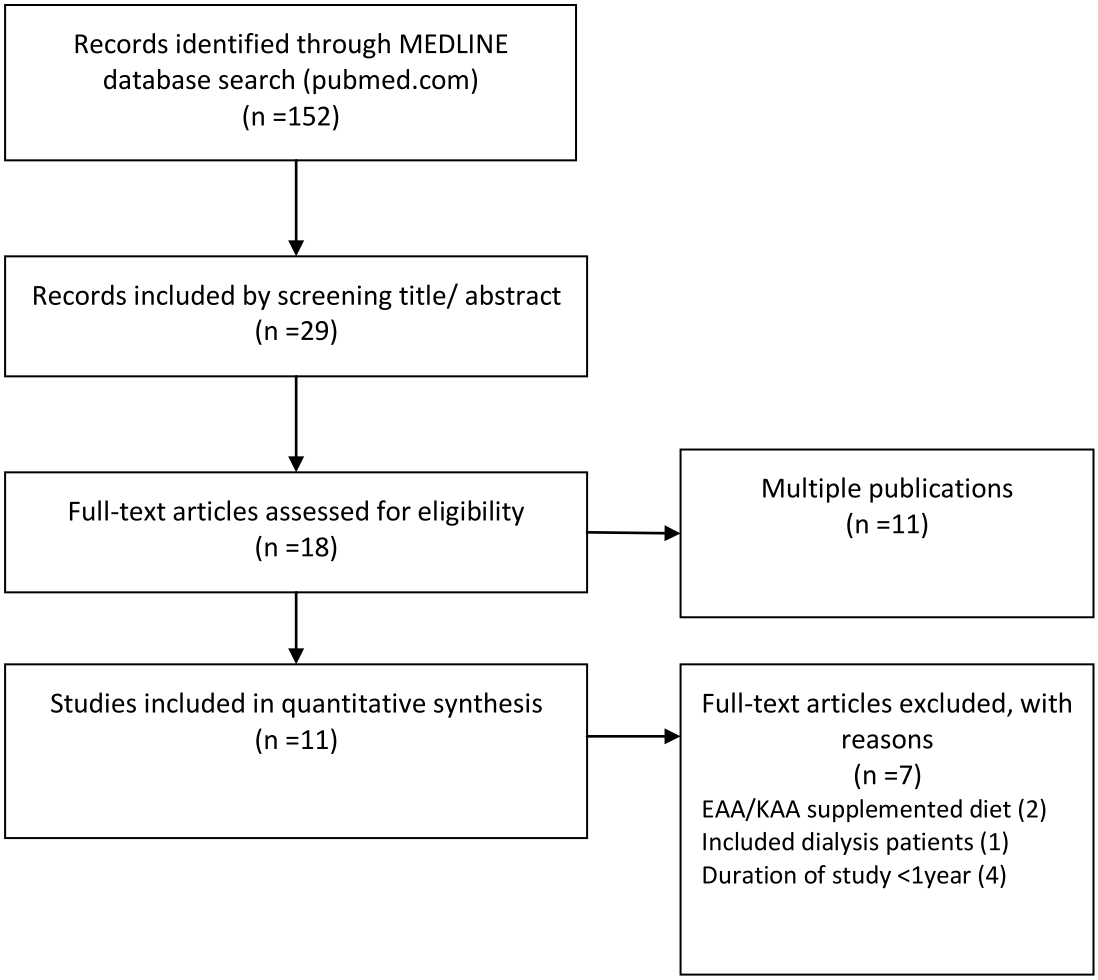

Supplement: S3 File — (PDF) [file pone.0145505.s003.pdf]
