# Supplementary material for: Protein Diet Restriction Slows Chronic Kidney Disease Progression in Non-Diabetic and in Type 1 Diabetic Patients, but Not in Type 2 Diabetic Patients: A Meta-Analysis of Randomized Controlled Trials Using Glomerular Filtration Rate as a Surrogate
Source: PLoS One. 2015 Dec 28;10(12):e0145505. doi: 10.1371/journal.pone.0145505 (PMC4692386; doi:10.1371/journal.pone.0145505)
Supplement: S4 File — (PDF) [file pone.0145505.s004.pdf]

Assessment of Bias using the Cochrane Risk of Bias tool

Table. Assessment of Bias using the Cochrane Risk of Bias tool [a].

Index	Author	RS	AC	Method of AC	BPar	BPer	BOA	IOD	SRB	Other [b]	OS [c]
1	B.U.Ihle et al.	low	unclear		high	high	unclear	low	low	low	unclear
2	B.H.Brouhard et al.	low	unclear		high	high	low	low	low	low	unclear
3	K.Zeller et al.	low	unclear		high	high	low	low	low	low	unclear
4	P.S.Williams et al.	low	low	cards and random numbers	high	high	low	low	low	low	low
5	R.P.F.Dullaart et al.	low	low	sealed envelopes	unclear	unclear	low	low	low	low	low
6	S.Klahr et al. StudyA	low	unclear		high	high	low	low	low	low	unclear
7	H.P.Hansen et al.	low	unclear		high	high	low	low	low	low	unclear
8	L.T.J.Pijls et al.	low	low	random numbers	low	low	low	low	low	low	low
9/10	C.Meloni et al.	low	low	random numbers	high	high	low	low	low	low	low
11	B.Dussol et al.	low	low	random numbers	high	high	low	low	low	low	low
12	D.Koya et al.	low	low	central	high	high	low	low	low	low	low
13	B.Cianciaruso et al.	low	low	sealed envelopes	unclear	unclear	low	low	low	high	high
14	N.R.Larsen et al.	low	low	random numbers	unclear	low	low	low	low	low	low
15	D.R.Jesudason et al.	low	low	sealed envelopes	high	high	low	low	low	high	high

[a] RS, Random Sequence; AC, Allocation Concealment; Method of AC, Method of Allocation Concealment; BPar, Blinding of Participants; BPer, Blinding of Personnel; BOA, Blinding of Outcome Assessment; IOD, Incomplete Outcome Data; SRB, Selective Reporting Bias; OS, Overall Score. Bias graded in 3 categories; namely low risk of bias, unclear risk of bias, and high risk of bias.

[b] Other risk of bias column graded as high or low in this category. The studies using the MDRD equation to derive estimated Glomerular Filtration Rate were rated as having high risk of bias. There were only two studies having high risk of bias, one of which included subjects with GFR< 60 ml/min/m^2.

[c] Overall score is the worst grade of bias in the individual domains. BPar and BPer were not considered for the overall scoring.

Figure. Bar chart to illustrate possible sources of bias using the Cochrane Risk of Bias tool [a].

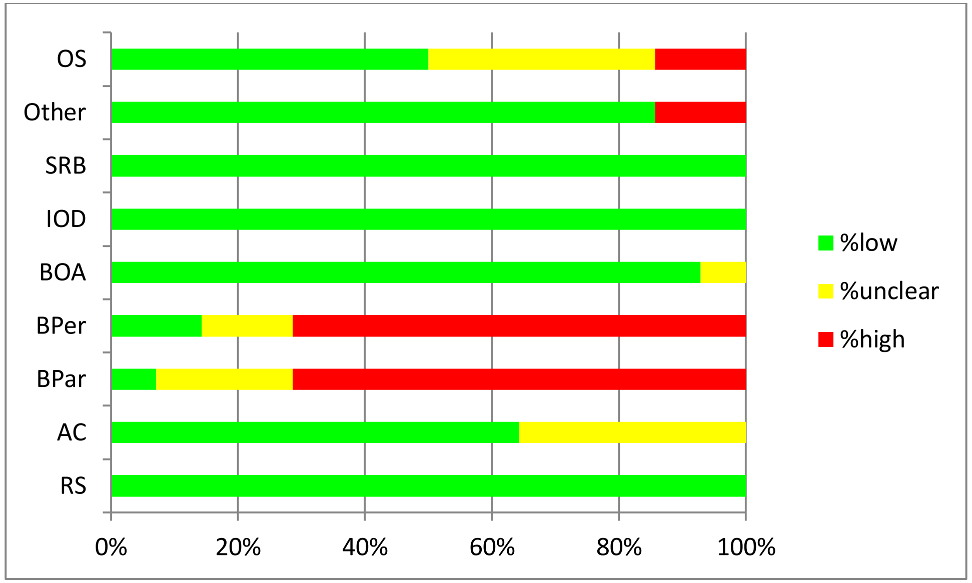

[a] RS, Random Sequence; AC, Allocation Concealment; Method of AC, Method of Allocation Concealment; BPar, Blinding of Participants; BPer, Blinding of Personnel; BOA, Blinding of Outcome Assessment; IOD, Incomplete Outcome Data; SRB, Selective Reporting Bias; OS, Overall Score
