# Supplementary figures and images for: Protein Diet Restriction Slows Chronic Kidney Disease Progression in Non-Diabetic and in Type 1 Diabetic Patients, but Not in Type 2 Diabetic Patients: A Meta-Analysis of Randomized Controlled Trials Using Glomerular Filtration Rate as a Surrogate
Source: PLoS One. 2015 Dec 28;10(12):e0145505. doi: 10.1371/journal.pone.0145505 (PMC4692386; doi:10.1371/journal.pone.0145505)

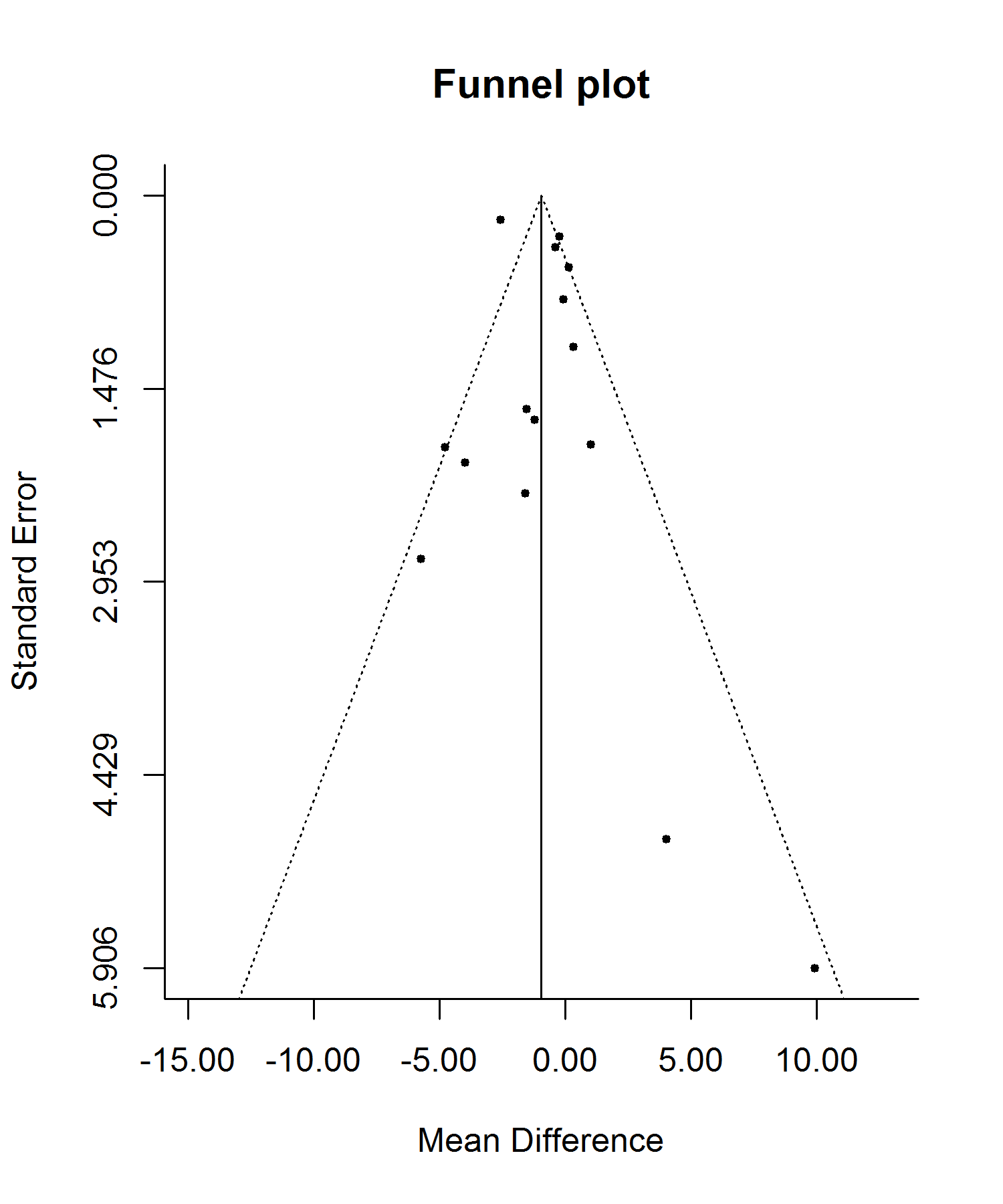

Supplement: S1 Fig — The plot is almost symmetrical with 7 studies on one side and 8 on the other. (TIF) [file pone.0145505.s006.tif]

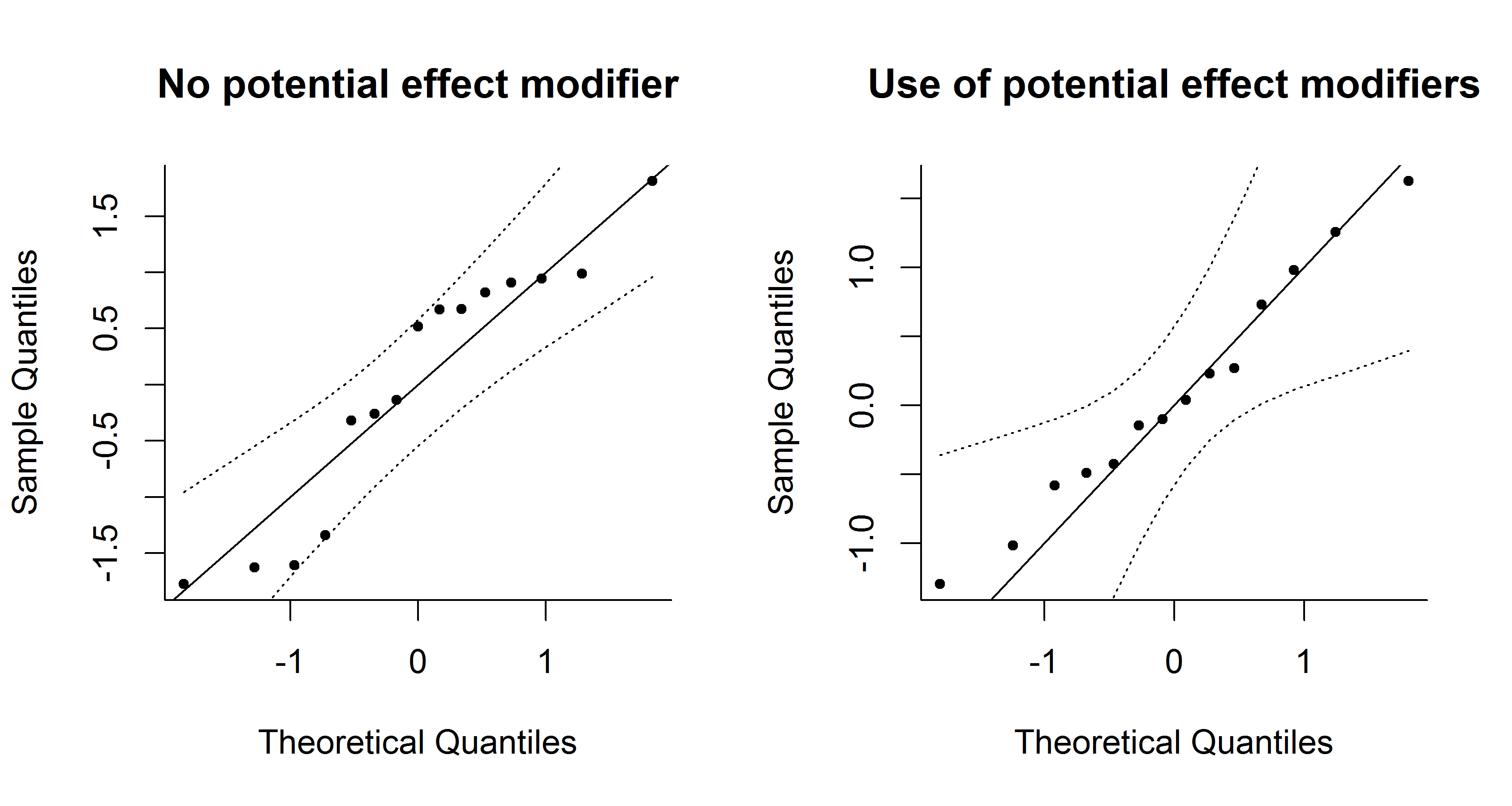

Supplement: S2 Fig — The left graph represents the probability plot for the random-effects model (no use of potential effect modifiers) and the right graph represents the random-effects meta-regression model (with use of the potential effect modifiers). The solid line represents the predicted effect for the normal distribution and the each dot represents a study. There is little organization in the distribution of dots in the random-effects model, with some close to the boundaries. In the plot for the random-effects meta-regression model with use of potential effect modifiers, it can be seen that the dots are more close to the predicted line. A normal distribution is achieved. The dots are arranged in a fairly straight line. (TIF) [file pone.0145505.s007.tif]
